# Biocatalysis and Bioactive Molecules: Future and Development

**DOI:** 10.3390/ijms24065571

**Published:** 2023-03-14

**Authors:** Daniela Remonatto, Lindomar Alberto Lerin

**Affiliations:** 1 Department of Bioprocesses and Biotechnology Engineering, School of Pharmaceutical Sciences, São Paulo State University (UNESP), Araraquara 4800-903, SP, Brazil; 2 Department of Chemistry, Pharmaceutical and Agricultural Sciences, University of Ferrara, Via Luigi Borsari 46, 44121 Ferrara, Italy

Due to the increasing interest in molecules obtained by bioprocesses over the past decade, biocatalysis has gained momentum in a variety of industrial sectors. Molecules obtained by enzymatic synthesis are high-added-value products recognized as natural agents. In addition, enzymatic reactions allow reductions in waste generation, thereby promoting sustainable development. However, to consolidate the use of these biocatalysts in industry, efforts are still necessary to develop biocatalysts with high stability, longer half-life time, and lower economic impact.

Currently, relevant topics within the biocatalysis field include directed evolution to improve the properties of enzymes, enzymes to perform new-to-nature reactions, and the development of green processes and technologies, mainly through valuing renewable resources.

Enzyme-improvement strategies that yield biocatalysts with better properties include the search for new methods/supports for enzyme immobilization and the use of protein engineering. When well designed, immobilization can be a very powerful tool to improve enzyme stability and widen the applicability of enzymes in industrial settings [1]. For this, the careful selection of the immobilization method and carrier matrix has fundamental importance. Through protein-engineering techniques, it is possible to alter enzyme properties, improve reaction performance metrics, and expand the application of biocatalysts to new reactions.

The needs that will define the future of biocatalysis include new types of enzyme-catalyzed reactions, the development of cost-effective enzyme synthesis routes to expand the use of enzymes in bioprocesses, and the search for enzymes with additional abilities, such as catalytically promiscuous ones that can expand the catalytic action to new reactions.

Biologically active molecules are natural compounds of particular interest to the pharmaceutical, food, and dermo-cosmetic industries. These molecules are usually chiral compounds produced by living organisms as single stereoisomers that cannot be artificially obtained. From this perspective, biocatalysis offers a broad range of easily accessible enzymes to perform challenging reactions with excellent results in terms of yield and selectivity [2]. Biocatalytically produced bioactive molecules can improve quality of life as they are environmentally friendly and also have beneficial impacts on the health of the human body in the prevention and treatment of various chronic diseases.

To achieve real progress in sustainability, a more circular production from renewable biomass is necessary. The use of alternative substrates in bioprocesses, such as agro-industrial waste, complements circular economy principles. Furthermore, to guarantee the development of green processes, other aspects must be observed, such as the use of biosolvents for the recovery and purification of biocompounds. Finally, high-quality environmental and economic assessments are increasingly seen as fundamental in evaluating the impacts of implementing new cost-effective processes.

Papers regarding biocatalysis and the bioactive molecules field are welcomed for submission to the current Special Issue entitled “Biocatalysis and Bioactive Molecules: Future and Development” to analyze and discuss processes aimed at the development of more sustainable synthesis routes.

## Data Availability

Not applicable.
